# Altered Interhemispheric Functional Coordination in Chronic Tinnitus Patients

**DOI:** 10.1155/2015/345647

**Published:** 2015-02-19

**Authors:** Yu-Chen Chen, Wenqing Xia, Yuan Feng, Xiaowei Li, Jian Zhang, Xu Feng, Cong-Xiao Wang, Yu Cai, Jian Wang, Richard Salvi, Gao-Jun Teng

**Affiliations:** ^1^Jiangsu Key Laboratory of Molecular and Functional Imaging, Department of Radiology, Zhongda Hospital, Medical School, Southeast University, Nanjing 210009, China; ^2^Center for Hearing and Deafness, University at Buffalo, State University of New York, Buffalo, NY 14214, USA; ^3^Medical School, Southeast University, Nanjing 210009, China; ^4^Center for Functional Neuroimaging, University of Pennsylvania, Philadelphia, PA 19104, USA; ^5^Department of Radiology, Nanjing First Hospital Affiliated to Nanjing Medical University, Nanjing 210006, China; ^6^Department of Otolaryngology, Zhongda Hospital, Medical School, Southeast University, Nanjing 210009, China; ^7^Department of Physiology, Southeast University, Nanjing 210009, China; ^8^School of Human Communication Disorders, Dalhousie University, Halifax, NS, Canada B3J 1Y6

## Abstract

*Purpose*. Recent studies suggest that tinnitus may be due in part to aberrant callosal structure and interhemispheric interaction. To explore this hypothesis we use a novel method, voxel-mirrored homotopic connectivity (VMHC), to examine the resting-state interhemispheric functional connectivity and its relationships with clinical characteristics in chronic tinnitus patients. *Materials and Methods*. Twenty-eight chronic tinnitus patients with normal hearing thresholds and 30 age-, sex-, education-, and hearing threshold-matched healthy controls were included in this study and underwent the resting-state fMRI scanning. We computed the VMHC to analyze the interhemispheric functional coordination between homotopic points of the brain in both groups. *Results*. Compared to the controls, tinnitus patients showed significantly increased VMHC in the middle temporal gyrus, middle frontal gyrus, and superior occipital gyrus. In tinnitus patients, a positive correlation was found between tinnitus duration and VMHC of the uncus. Moreover, correlations between VMHC changes and tinnitus distress were observed in the transverse temporal gyrus, superior temporal pole, precentral gyrus, and calcarine cortex. *Conclusions*. These results show altered interhemispheric functional connectivity linked with specific tinnitus characteristics in chronic tinnitus patients, which may be implicated in the neuropathophysiology of tinnitus.

## 1. Introduction

Chronic subjective tinnitus, a phantom auditory perception that occurs in the absence of an acoustic source [1, 2], affects around 5–15% of adults. In severe cases, chronic tinnitus can lead to sleep disturbances, inability to concentrate, work impairment, and emotional distress [3, 4]. Current theories assume that chronic tinnitus arises from maladaptive neuroplastic changes in the central nervous system rather than the cochlea since tinnitus often persists even after sectioning the auditory nerve [5, 6]. A variety of pathophysiological changes have been postulated to give rise to tinnitus, such as increased burst firing, enhanced neural synchrony, increased spontaneous activity, tonotopic map reorganization, abnormal coupling of networks involving auditory and nonauditory structures, and aberrant consciousness gating mechanisms [7]. Despite extensive study, the neuropathophysiological mechanisms responsible are not fully understood.

Because of the multidimensional nature of tinnitus, there is growing recognition that tinnitus may result from abnormal coupling between different functional centers. Using resting-state functional magnetic resonance imaging (fMRI) of the blood-oxygenation-level dependent (BOLD) time series, several studies demonstrated aberrant functional connectivity among auditory and nonauditory brain areas in tinnitus patients [8]. Using seed-based functional connectivity analysis, negative correlations were observed between BOLD responses in auditory cortex and visual, attention, and control networks in bothersome tinnitus [9]. Furthermore, multiple brain resting-state networks, such as the default mode network (DMN) and dorsal attention network (DAN), were disrupted in tinnitus patients providing support for aberrant neural coupling of diverse brain regions [10–13].

The corpus callosum (CC), the largest commissural fiber bundle, facilitates communication and integration of emotional, cognitive, motor, and sensory information between the two cerebral hemispheres [14, 15]. Despite its importance, there is a dearth of information regarding changes in interhemispheric functional connectivity in tinnitus patients. However, recent MRI studies have identified interhemispheric structural changes in tinnitus patients [16–19]. Diesch and colleagues demonstrated larger volume of the posterior CC splenium in tinnitus patients [16]. Since many auditory fibers cross in the posterior part of the CC [20], the increased volume might be indicative of stronger interhemispheric connectivity. Diffusion tensor imaging (DTI) identified alterations in white matter (WM) integrity in the CC of tinnitus patients [18, 19]. Aldhafeeri et al. suggested that abnormal interhemispheric signaling could arise from an imbalance in excitation and inhibition in the two hemispheres [19]. Abnormalities in the CC could impair interhemispheric functional interactions that are fundamental to integrative attentional processing and executive control [21, 22], consistent with the disruptions in attentional and executive control networks observed in tinnitus patients [8, 9, 12]. Taken together, these results suggest that it could be informative to compare the interhemispheric functional coordination in tinnitus patients and matched controls to determine if there are major differences.

To address this issue, we evaluated the interhemispheric functional coordination in patients with chronic tinnitus using voxel-mirrored homotopic connectivity (VMHC), a novel method for evaluating on resting-state fMRI [23]. VMHC is a voxelwise measurement of functional homotopy that reveals the synchrony of resting-state functional connectivity between a voxel in one hemisphere and its mirrored counterpart in the other. VMHC has been successfully used to explore the interhemispheric functional coordination in autism, schizophrenia, cocaine addiction, and other diseases [24–28]; however, VMHC has, to our knowledge, not been used previously to investigate interhemispheric functional coordination in tinnitus patients and to study the relationships between altered interhemispheric functional connectivity and other tinnitus characteristics such as tinnitus duration or severity.

## 2. Materials and Methods

### 2.1. Subjects

All the subjects provided written informed consent before their participation in the study protocol, which was approved by the Research Ethics Committee of the Affiliated Zhongda Hospital of Southeast University.

This study was conducted from September 2011 to September 2013. A total of 59 subjects including 29 chronic tinnitus patients and 30 healthy controls were recruited through community health screening and newspaper advertisements. The tinnitus patients and healthy subjects were group-matched in terms of age, sex, and education. One tinnitus patient was subsequently excluded because the limits for head motion were exceeded during MR scanning. Twelve patients reported a predominantly left-sided tinnitus, nine patients reported a predominantly right-sided tinnitus, and seven patients described their tinnitus as bilateral or originating within the head. All subjects were right-handed and completed at least 8 years of education. The patients were between 25 and 64 years of age (40.5 ± 13.2 years), with tinnitus duration between 6 and 120 months (34.3 ± 34.2 months). The severity of tinnitus and related distress were assessed by the Iowa version of the Tinnitus Handicap Questionnaires (THQ) [29] which has a three-factor structure. Factor 1 reflects the social, emotional, and physical consequences of tinnitus, factor 2 the hearing ability of the patient, and factor 3 the patients' view of tinnitus. Hearing thresholds were determined by pure tone audiometry (PTA). All of the participants had normal hearing (defined as thresholds <25 dB HL) at the 10 measured audiometric frequencies ranging from 250 Hz to 16 kHz. There were no significant differences in auditory thresholds between tinnitus and control groups. In addition, none of the participants had symptoms of depression and anxiety according to the Self-Rating Depression Scale (SDS) and Self-Rating Anxiety Scale (SAS) (overall scores <50, resp.) [30, 31].

Participants were excluded from the study if they suffered from hyperacusis, pulsatile tinnitus, or Meniere's diseases or if they had a past history of severe smoking, stroke, alcoholism, brain injury, Parkinson's disease, Alzheimer's disease, epilepsy, major depression, neurological or psychiatric disorders that could affect cognitive function, major medical illness (e.g., anemia, thyroid dysfunction, and cancer), MRI contraindications, or severe visual loss. The characteristics of the chronic tinnitus patients and healthy subjects are summarized in Table 1.

### 2.2. MRI Acquisition

MRI data were acquired at the Radiology Department of Zhongda Hospital using a 3.0 T MRI scanner (Siemens MAGENETOM Trio, Erlangen, Germany). Head motion and scanner noise were reduced using foam padding and earplugs. The earplugs (Hearos Ultimate Softness Series, USA) were used to attenuate scanner noise by approximately 32 dB. Subjects were instructed to lie quietly with their eyes closed without falling asleep, not think of anything in particular, and avoid any head motion during the scan. Functional images were obtained axially using a gradient-echo-planar imaging sequence as follows: repetition time (TR) = 2000 ms; echo time (TE) = 25 ms; slices = 36; thickness = 4 mm; gap = 0 mm; field of view (FOV) = 240 mm × 240 mm; acquisition matrix = 64 × 64; and flip angle (FA) = 90°. The images were acquired in an interleaved order. The fMRI sequence took 8 minutes and 6 seconds. Structural images were acquired with a T1-weighted 3D spoiled gradient-echo sequence as follows: TR = 1900 ms; TE = 2.48 ms; slices = 176; thickness = 1 mm; gap = 0 mm; FA = 90°; acquisition matrix = 256 × 256; and FOV = 250 mm × 250 mm. The structural sequence took 4 minutes and 18 seconds.

### 2.3. Functional Data Preprocessing

Functional data analyses were conducted using Data Processing Assistant for Resting-State fMRI (DPARSF) programs [32] based on statistical parametric mapping (SPM8, http://www.fil.ion.ucl.ac.uk/spm/) and resting-state fMRI data analyses toolkits (REST, http://www.restfmri.net/). A total of 240 volumes were scanned, and the first 10 volumes were discarded to allow for signal equilibrium of the initial magnetic resonance signals and adaptation of the subjects to scanner. The remaining 230 consecutive volumes were used for data analysis. Afterwards, the following procedures were carried out as follows: slice-timing adjustment, realignment for head motion correction, spatial normalization to the Montreal Neurological Institute (MNI) template (resampling voxel size = 3 × 3 × 3 mm^3^) and smoothing with an isotropic Gaussian kernel (FWHM = 6 mm), and detrending and filtering (0.01–0.08 Hz). Any subjects with a head motion >2.0 mm translation or a 2.0° rotation in any direction were excluded.

### 2.4. VMHC Analyses

The VMHC computation was processed using REST software. As described previously [23], the homotopic functional connectivity was computed as Pearson's correlation coefficient between the residual time series of each voxel and that of its symmetrical interhemispheric counterpart. Afterwards, the correlation values were Fisher z-transformed to improve the normality of the values. The resultant values were referred to as VMHC and were used for the group analyses.

### 2.5. Structural Data Analyses

To exclude the possibility of structural differences on VMHC measurements, we performed a voxel-based morphometry (VBM) approach to compute the gray matter (GM) volume and WM volume of each subject using DPARSF software. Briefly, cerebral tissues were segmented into GM, WM, and cerebrospinal fluid and were then normalized to the MNI space using a unified segmentation algorithm [33]. T1 images were normalized to the MNI template using affine linear registration followed by Gaussian smoothing (FWHM = 6 mm). GM and WM volumes were calculated by estimating these segments. Brain parenchyma volume was calculated as the sum of GM and WM volumes. The normalized GM images of all subjects were averaged and the generated mean image was averaged with its left-right mirror to get the symmetrical template and mask for VMHC and statistical analyses.

### 2.6. Statistical Analysis

Differences in demographic data between tinnitus patients and healthy controls were analyzed using between-group *t*-test for means and *χ* ^2^-test for proportions (statistical significance was set at *P* < 0.05).

Individual VMHC maps were entered into a voxelwise two-sample *t*-test to examine the differences in interhemispheric functional connectivity between groups. The modulated GM maps obtained from the VBM analysis were applied to exclude the possible effects of structural differences. The statistical threshold was set at corrected *P* < 0.05 (combination of *P* < 0.01 for single voxel and a minimum cluster size of 40 voxels), which was determined by Monte Carlo simulations using the AFNI AlphaSim program (http://afni.nimh.nih.gov/pub/dist/doc/manual/AlphaSim.pdf). Age, sex, and education were included as nuisance covariates.

To investigate the relationship between the VMHC and tinnitus characteristics, including the tinnitus duration and distress, Pearson correlation analyses were performed in a voxelwise manner using REST software. The statistical threshold was set at corrected *P* < 0.05 using the same parameters as the group comparison analysis of VMHC.

Since micromovements from volume to volume can influence the functional connectivity [34], framewise displacement (FD) values were computed for each subject to reflect the temporal derivative of the movement parameters. These were used as covariates in the group level analyses of VMHC. No subjects had FD > 0.5 mm on greater than 35 volumes in this study. No significant difference was found in the mean FD values between tinnitus patients (0.22 ± 0.06 mm) and controls (0.20 ± 0.06 mm) in the final sample (*t*-tests *t* = 1.23, *df* = 56, *P* = 0.224).

## 3. Results

### 3.1. Interhemispheric Connectivity Differences

**Figure 1:**
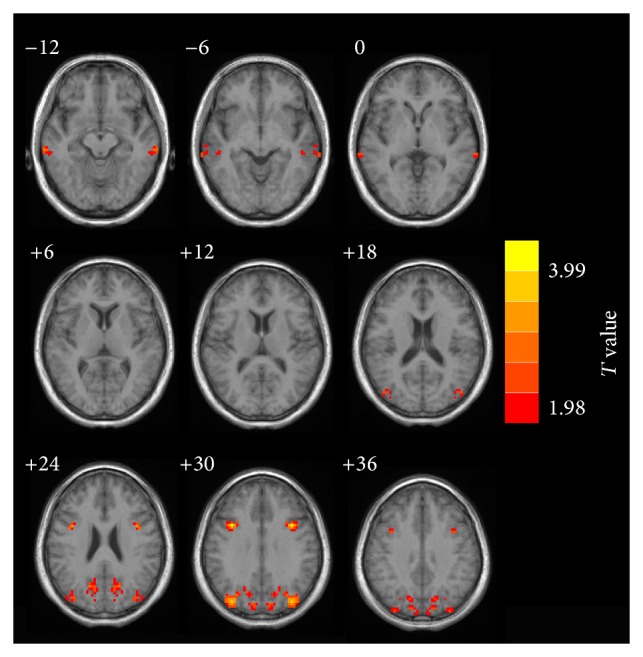
The regions which showed significantly increased VHMC values between tinnitus patients and healthy controls. Thresholds were set at a corrected *P* < 0.05, determined by Monte Carlo simulation. Note that the left side corresponds to the right hemisphere.

Compared with the healthy controls, the tinnitus patients showed significant increases in VMHC in several brain regions, namely, the middle temporal gyrus (MTG), middle frontal gyrus (MFG), and superior occipital gyrus. No significant decreases in VHMC were observed in tinnitus patients compared to controls (Figure 1 and Table 2).

### 3.2. Correlation Analysis Results

**Figure 2:**
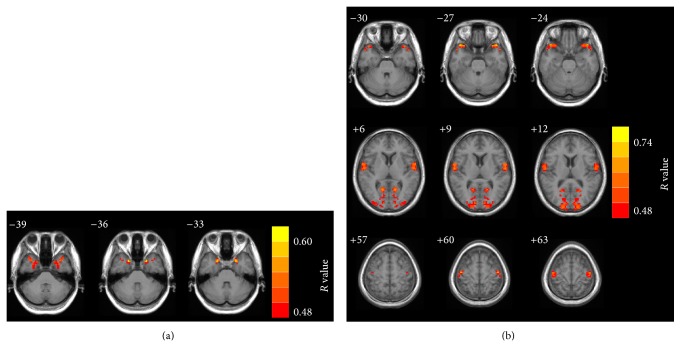
Regions showing significant correlations between VMHC and tinnitus characteristics. (a) Significant positive correlation between the tinnitus duration and VMHC in the uncus. (b) Significant positive correlations between the THQ score and VMHC in the transverse temporal gyrus, the superior temporal pole, the precentral gyrus, and the calcarine cortex. Thresholds were set at a corrected *P* < 0.05, determined by Monte Carlo simulation. Note that the left side corresponds to the right hemisphere.

Voxelwise correlation analyses revealed a significant positive correlation between the tinnitus duration and VMHC in the uncus, an anterior extremity of the parahippocampal gyrus. Significant positive correlations were also found between the THQ scores and VMHC in the transverse temporal gyrus, superior temporal pole, precentral gyrus, and calcarine cortex (Figure 2 and Table 3). These correlations survived after correction for age, sex, and education.

### 3.3. Structural Analysis Results

Table 4 presents the comparisons of the whole brain volumes (GM volume, WM volume, and brain parenchyma volume) between the tinnitus patients and the healthy subjects. No significant differences in GM and WM volumes were found between tinnitus patients and the control group (*P* > 0.05). Moreover, no significant differences between patients and controls were found in the GM volumes of the abovementioned regions showing altered VMHC.

## 4. Discussions

To our knowledge, this is the first study to use VMHC to identify changes in interhemispheric functional connectivity and to correlate these changes to specific features of tinnitus. Using VMHC, we found that interhemispheric connectivity was significantly enhanced in selected brain regions, namely, the MTG, MFG, and visual cortex. Tinnitus distress was positively correlated with VMHC in auditory, motor, and visual cortices. On the other hand, tinnitus duration was positively correlated with VMHC in parahippocampal regions.

Interhemispheric connectivity was significantly enhanced in the MTG of tinnitus patients compared to matched controls. These results are somewhat surprising given that previous structural MRI studies have observed reductions of GM volume and cortical thickness bilaterally in the MTG (Brodmann area 21) of tinnitus patients compared to controls [19, 35, 36]. Since we did not observe a significant decrease in GM volume between tinnitus patients and controls, the increased interhemispheric connectivity seen in tinnitus patients is unlikely due to structural differences but rather enhanced neural activity, synaptic coupling, or synchrony between these regions. Consistent with this view, Mirz et al. found increased neuronal activity in the right hemisphere of tinnitus patients with a focus on the MTG using positron emission tomography (PET) imaging [37]. Moreover, using resting-state fMRI, we found increased amplitude of low-frequency fluctuation in the right MTG of chronic tinnitus patients [38]. The increased activity in the right MTG of tinnitus patients could enhance the functional coordination between the left and right MTG in tinnitus patients thereby raising the perceptual awareness of neural activity in this region. The MTG is an important component of the DMN, a network activated when individuals engage in internal activities such as dreaming or memory recall [39, 40]. The increased interhemispheric connectivity in bilateral MTG might reflect dysfunctions of the DMN linked to tinnitus as in previous resting-state fMRI studies [9, 10, 41].

The frontal cortex is thought to play a pivotal role in tinnitus [42], and consistent with this view we found increased interhemispheric connectivity in MFG. Neuroimaging studies have identified abnormalities in the frontal cortex associated with tinnitus [38, 43, 44]. Patients with high and low tinnitus distress showed significant differences in the amount of fMRI activation in the left MFG when listening to tinnitus related sentences [45]. Aldhafeeri et al. reported alterations of cortical thickness in bilateral MFG in patients with moderate to high scores on tinnitus intrusiveness and severity [19]. Moreover, in tinnitus patients, Diesch et al. detected enhanced fiber projections through the anterior CC [16], a region which carries fiber projections of prefrontal, premotor, and supplementary motor cortex [46]. Enhanced VMHC in the MFG could reflect the enhanced negative emotions associated with tinnitus [47]. Likewise, the altered anterior CC may account for the correlation between the tinnitus distress and the VMHC in the precentral gyrus, a crucial brain region showing aberrant neuronal activity in tinnitus patients [11, 45, 48].

We also observed increased VMHC in the superior occipital gyrus as well as a positive correlation between the tinnitus distress and VMHC in the calcarine cortex. Cate et al. found that auditory attention could activate peripheral visual cortex [49]. One interpretation of these results is that as patients attend to their phantom auditory sensation they contemporaneously activate visual areas. This interpretation is consistent with previous fMRI studies showing aberrant function in visual network in tinnitus patients [9, 41].

Interestingly, we observed a positive correlation between tinnitus duration and VMHC in the uncus, located in the anterior part of the parahippocampal gyrus and considered as a part of the limbic system. In fMRI studies, the uncus was reported to respond weakly to speech stimulation and masking noise suppressed the response to speech stimuli [50]. The parahippocampal area has been hypothesized to play a central role in memory recollection and transferring information from the hippocampus to the association areas [51]. Electroencephalography (EEG) study suggested that tinnitus patients differed from healthy controls by increased delta and theta activity in the parahippocampal gyrus [52]. Increased functional connectivity was found between the auditory cortices and the left parahippocampus in tinnitus patients providing further support linking tinnitus with limbic areas [41]. Recently, Schecklmann et al. confirmed that tinnitus distress correlated positively with activation of left and right posterior parahippocampal-hippocampal interface revealed by PET imaging [44]. Our correlation results presumably indicate the interhemispheric functional coordination deficit in limbic system due to tinnitus.

In addition to the parahippocampus, we mainly observed significant positive correlations between the VMHC and tinnitus distress in the primary auditory cortex. Previous MRI studies on tinnitus have reported decreased or increased GM volume in primary auditory cortex [17, 19, 53]. Arguing that the volume of the CC relative to the volume of auditory cortex might be a better measure of the connectivity between auditory cortices, Diesch et al. found enlarged posterior CC splenium (the CC area where the interhemispheric auditory pathways cross) in tinnitus patients [16]. Moreover, subjective intrusiveness of tinnitus was correlated positively with the size of the posterior part of CC. Thus, it could be hypothesized that stronger interhemispheric connectivity between auditory cortices might underlie the neuropathological mechanisms of tinnitus.

Previous studies have reported decreased or increased GM volume in tinnitus patients in both auditory and nonauditory regions including Heschl's gyrus [19, 36, 53], superior temporal gyrus [17, 19, 54], inferior colliculus [55], anterior cingulate cortex [17, 19], ventromedial prefrontal cortex [19, 54, 56], nucleus accumbens [57], hippocampus [53, 55], and occipitoparietal cortex [53]. Nonetheless, the changes in GM volume seen in these tinnitus patients were typically linked with hearing loss particularly when testing was extended out beyond 8 kHz [47, 53, 58, 59]. Unlike these reports, we failed to detect significant differences in whole brain GM volume as well as regional GM volume in any of the regions showing altered VMHC in tinnitus patients. A probable explanation for this is the absence of any hearing loss out to 16 kHz and the absence of hyperacusis in our normal hearing tinnitus patients. However, an alternative possibility is that our analytical techniques are not sensitive enough to detect the brain structural alterations in our tinnitus patients [53, 60]. Thus, our results suggest that altered interhemispheric functional connectivity can exist prior to any obvious structural changes in tinnitus patients with normal hearing and absence of hyperacusis.

Several practical constraints should be noted in our study. First, considering the cross-sectional nature of our experimental design and limited sample size (~30/group), further studies are needed with a larger and different population of subjects. Alternatively, longitudinal studies incorporating therapies to alter the tinnitus characteristic could be used to examine the relationship between interhemispheric functional coordination and tinnitus characteristics. Second, as noted above, impairment in the WM integrity, particularly in the CC, might shed light on the role structural changes may play in VMHC changes in tinnitus. Besides measuring WM volume, a more informative approach would be to use DTI to detect changes in anisotropy within specific fiber tracts relevant to VMHC. Another confounding factor for all resting-state fMRI studies is scanner noise that activates auditory pathways to varying degree [61]; however, this confound should affect our normal hearing tinnitus patients and controls to a similar degree. Finally, Gu et al. demonstrated that subjects with hyperacusis showed enhanced activation in the auditory midbrain, thalamus, and the primary auditory cortex [62]. We attempted to exclude subjects with hyperacusis from our study. However, future studies employing tinnitus patients with and without hyperacusis could help distinguish those regions of the brain associated with hyperacusis and tinnitus versus tinnitus alone. Since tinnitus subjects with normal hearing thresholds represent a small subset of all tinnitus patients, a tinnitus subgroup with hearing loss would be useful to be included in future studies.

## 5. Conclusions

Our results show that increased interhemispheric functional coordination revealed by VMHC is present in chronic tinnitus in patients with normal hearing, without hyperacusis and changes in GM and WM volumes. Enhanced VMHC in the anterior parahippocampal gyrus (uncus) was positively correlated with tinnitus duration, whereas THQ scores were positively correlated with VMHC in the transverse and superior temporal gyrus, precentral gyrus, and visual cortex. VMHC analysis might provide a novel approach to evaluating therapeutic approaches aimed at ameliorating abnormal functional connections in brain regions associated with tinnitus and tinnitus distress.

## Figures and Tables

**Table 1:** Characteristics of the tinnitus patients and healthy controls.

	Tinnitus patients	Healthy controls	*P* value
	(*n* = 28)	(*n* = 30)	
Age (year)	40.5 ± 13.2	46.2 ± 11.9	0.090
Gender (male : female)	16 : 12	15 : 15	0.586
Education levels (years)	10.8 ± 1.6	11.1 ± 1.7	0.545
Tinnitus duration (months)	34.3 ± 34.2	—	—
THQ total score	41.3 ± 18.2	—	—
Factor 1	45.0 ± 22.2	—	—
Social subscale	11.5 ± 6.2	—	—
Emotional subscale	17.0 ± 10.6	—	—
Physical subscale	16.5 ± 8.0	—	—
Factor 2	28.1 ± 26.0	—	—
Factor 3	54.1 ± 17.0	—	—

Data are represented as mean ± SD.

**Table 2:** Regions showing significant differences in VMHC between tinnitus patients and healthy controls.

Brain regions	BA	Peak MNI coordinates	Peak *T* value	Voxels
		*x*, *y*, *z* (mm)		
Middle temporal gyrus	21	±69, −30, −12	3.1750	50
Middle frontal gyrus	11	±36, 12, 30	4.3978	48
Superior occipital gyrus	19	±36, −81, 30	3.8459	102

A corrected threshold of *P* < 0.05 determined by Monte Carlo simulation was taken as meaning that there was a significant difference between groups. BA, Brodmann's area; MNI, Montreal Neurological Institute.

**Table 3:** Regions showing significant correlations between VMHC and tinnitus characteristics.

Brain regions	BA	Peak MNI coordinates	Peak *R* value	Voxels
		*x*, *y*, *z* (mm)		
(I) VMHC and tinnitus duration				
uncus	28	±21, 6, −33	0.62026	68
(II) VMHC and THQ score				
Transverse temporal gyrus	42	±60, −9, 9	0.63775	194
Superior temporal pole	38	±42, 21, −27	0.71195	76
Precentral gyrus	4	±42, −15, 60	0.64225	48
Calcarine cortex	30	±15, −57, 6	0.65234	254

A corrected threshold of *P* < 0.05 was determined by Monte Carlo simulation. BA, Brodmann's area; MNI, Montreal Neurological Institute.

**Table 4:** Comparisons of the brain volumes between the tinnitus patients and healthy controls.

	Tinnitus patients	Healthy controls	*P* value
	(*n* = 28)	(*n* = 30)	
Gray matter	580.36 ± 27.02	573.40 ± 23.15	0.296
White matter	530.75 ± 25.45	528.13 ± 25.69	0.698
Brain parenchyma	1111.11 ± 32.18	1101.53 ± 37.21	0.300

Data are presented as mean ± SD.
